# Ethnobotany of dye plants in Dong communities of China

**DOI:** 10.1186/1746-4269-10-23

**Published:** 2014-02-19

**Authors:** Yujing Liu, Selena Ahmed, Bo Liu, Zhiyong Guo, Weijuan Huang, Xianjin Wu, Shenghua Li, Jiangju Zhou, Qiyi Lei, Chunlin Long

**Affiliations:** 1 College of Life and Environmental Sciences, Minzu University of China, Beijing 100081, China; 2 Sustainable Food and Bioenergy Systems Program, Department of Health and Human Development, Montana State University, Bozeman, MT 59717, USA; 3 Key Laboratory of Hunan Province for Study and Utilization of Ethnomedicinal Plant Resources, Huaihua University, Hunan 418000, China; 4 Kaili University, Guizhou 556011, China; 5 Kunming Institute of Botany, Chinese Academy of Sciences, Kunming 650201, China

**Keywords:** Dong people, Dye plant, Ethnobotany, Medicinal value

## Abstract

**Background:**

Dyes derived from plants have an extensive history of use for coloring food and clothing in Dong communities and other indigenous areas in the uplands of China. In addition to use as coloring agents, Dong communities have historically utilized dye plants for their value for enhancing the nutritive, medicinal and preservative properties of foods. However, the persistence of plant-derived dyes and associated cultural practices and traditional knowledge is threatened with rapid socio-economic change in China. Research is needed to document the ethnobotany of dye plants in indigenous communities towards their conservation and potential commercialization as a sustainable means of supporting local development initiatives.

**Methods:**

Semi-structured surveys on plants used for coloring agents and associated traditional knowledge were conducted in fifteen Dong villages of Tongdao County in Hunan Province of South Central China during 2011–2012. Transect walks were carried out with key informants identified from semi-structured surveys to collect samples and voucher specimens for each documented plant species for taxonomic identification.

**Results:**

Dong households at the study sites utilize the flowers, bark, stems, tubers and roots of 13 plant species from 9 families as dyes to color their customary clothing and food. Out of the documented plants, a total of 7 are used for coloring food, 3 for coloring clothing and 3 for both food and clothing. Documented plants consist of 3 species that yield black pigments, 3 for brownish red/russet pigments, 3 for red pigments, 2 for dark blue pigments and 2 for yellow pigments. In addition to dyes, the plants have multiple uses including medicinal, ornamental, sacrificial, edible, and for timber.

**Conclusions:**

The use of dyes derived from plants persists at the study sites for their important role in expressing Dong cultural identity through customary clothing and food. Further research is needed to evaluate the safety of dye plants, their efficacy in enhancing food items and their commercial potential. Conservation policies and management plans are called for to preserve these ethnobotanical resources in a sustainable manner that supports local livelihoods while maintaining cultural practices.

## Background

Dyes derived from plants have an extensive history of use for enhancing food and clothing in communities worldwide. Natural pigments have almost been completely replaced with synthetic dyes since the production of artificial dyes started in 1856 following synthesis by William Henry Perkin [1]. Artificial dyes are valued for their relatively cheaper prices, abundance and bright colors. However, artificial dyes are also recognized for their toxicity, contribution to environmental pollution and for their carcinogenic properties and can cause allergic reactions in humans [2]. Natural dyes on the other hand are recognized for their lower toxicity for both humans and the environment as well as being resilient to washing and fading [2,3]. Furthermore, some natural dyes are recognized for their positive health benefits such as the pigment uranidin from *Carthamus tinctorius*[2] and curcumin from the dried rhizomes of *Curcuma longa*[4]. The uranidin has been shown to accelerate blood circulation and promote skin metabolism [2], while curcumin is with antitumor, anti-inflammatory and antioxidant effects and it has been used for the treatment of rheumatism and prevent the generation of gallstones [4].

Some indigenous communities in remote areas continue to utilize dye plants for coloring food products and clothing as well as for other cultural uses. A literature review reveals that 88 patents have been filed for natural pigments from plants and that indigenous communities throughout the world utilized most of these resources [5]. Ethnobotanical research is needed to compile a comprehensive database of dye plants as well as to record associated knowledge and cultural practices for the preservation of these ethnobotanical resources. The present study seeks to address the knowledge gap through research in indigenous Dong communities in the uplands of South Central China.

Dong communities in the uplands of Central China have historically colored their cultural clothing and food products with dye plants and also used dye plants for adding medicinal and nutritive value to foods. The Dong socio-linguistic group is one of the 55 minority socio-linguistic groups of China. They primarily live in China’s Guizhou, Hunan and Guangxi Provinces. According to their language, Dong means "people who live inside of the fence". The Dong people are polytheism and animism. They worship natural objects including ancient trees and rocks as well as manmade objects including wells and bridges. Many Dong communities live in remote areas where there traditional practices persist. However, with expanded infrastructure and commercialization into China’s rural montane areas, traditional use of plants including dye plants is decreasing along with their associated knowledge base. The present study seeks to document dye plants in Dong communities and associated traditional knowledge towards cultural and ecological conservation.

## Methods

### Study sites

Research was conducted in Tongdao Dong Autonomous County in southwest Hunan Province of China. Tongdao County is one of the main settlements for the Dong that account for 78.3% of the total population of approximately 220,000 people. The county is 2,239 square kilometers in size with a total of 218 villages. The remaining population of Tongdao County includes 21.7% Han Chinese and small percentage of members from 13 other minority socio-linguistic groups. The mountains of Tongdao have historically served to create barriers restricting the supply and transport of goods and cultural exchange and enabled the persistence of Dong cultural practices. China’s recent efforts to expand infrastructure into its remote areas is now causing a notable shift in these communities.

Field research was carried out in Tongdao County in 2011 and 2012 in a total of 15 villages located in four townships including: Yutou, Yazhai, Ganzi, Tangchong, Jili, Luowu, Pipa, Qiaotou, Majia, Dazhai, Changzhai, Zhaishang, Hongxiang, Shangtuan and Lanyang villages. The population of these villages varies from 242 to 1,187. Two of the villages are close to a town (within five kilometers) while the others are far from township sites (25–35 kilometers).

### Field research

Semi-structured interviews were carried out at the study sites. A total of 80 informants including 36 males and 44 females were interviewed. Informants were between the ages of 16 and 70 years old. The interviews consisted of the following questions adapted from Wang and Long (1995) [6]: (1) What plants in your community have been traditionally used for dyeing products? (2) Who in your household and community uses dye plants? (3) What season/time of the year do you collect dye plants? (4) How are each of these dye plants collected? (5) Where do dye plants grow in your community and surroundings? (6) How are dye plants processed for prepared for coloring food and clothing? (7) Do you prefer your clothing to be from natural dyed cloth or artificially dyed cloth?

Fifteen key informants were identified on the basis of semi-structured interviews for transect walks through the surrounding mountains and fields to collect documented dye plants for samples and voucher specimens. Picture cards with dye plants were shown to informants to document local knowledge of these species.

### Data analysis

Dye plants reported from interviews were organized into a database (Table 1) with the following information: (1) Latin name, (2) family name, (3) parts used, (4) dye uses, (5) color and, (6) medicinal value. All the specimens were identified according to the *Flora of China*. Voucher specimens of plants available during field investigations were collected and deposited in the herbarium of Minzu University of China.

**Table 1:** Natural plants used for dying by Dong communities of Tongdao County in South Central China

**Herbarium Voucher #**	**Scientific name**	**Family name**	**Local Han-Chinese name**	**Dong name**	**Parts used**	**Dye uses**	**Color produced**	**Ecological data**	**Medicinal value**	**Other value**
Yujing Liu, D6 (MUC)	*Ardisia crenata* Roxb.	Myrsinaceae	Zhu sha gen朱砂根	Sang mei zhu	Root	Food and cloth	Red	Shrub under forest, wild	Curing throat swelling and pain, anti-fertility, antivirus, antineoplastic, insect disinfestations	Medicinal, ornamental
Yujing Liu, D13 (MUC)	*Adinandra milletii* Benth. et Hook. f. ex Hance	Theaceae	Yang tong杨桐	Mei tong wu	Leaves	Food	Black	Tree in forests, wild	None	Sacrifice
Yujing Liu, D4 (MUC)	*Buddleja lindleyana* Fortune	Loganiaceae	Zui yu cao 醉鱼草	**–**	Flower	Food	Yellow	Shrub on roadside or river side, wild	Anti-inflammatory, analgesic calm and protecting liver	Medicinal, ornamental
Yujing Liu, D3 (MUC)	*Buddleja officinalis* Maxim.	Loganiaceae	Mi meng hua密蒙花	**–**	Flower	Food	Yellow	Shrub on roadside or river side, wild	Treating dry eyes and gum (in the eyes) caused by liver vacuity, crying, blurred vision and cataracts caused by wind. It has also shown anti-inflammatory and blood glucose-reducing activity, as well as ability to strengthen immunity	Medicinal, edible
Yujing Liu, D8 (MUC)	*Dioscorea cirrhosa* Lour.	Dioscoreaceae	Shu liang薯莨	**–**	Tuber	Food	Brownish red	Mountain slope, wild	None	Medicinal, edible
Yujing Liu, D7 (MUC)	*Diospyros kaki* Thunb.	Ebenaceae	Shi 柿	Men	Bark	Food and cloth	Russet	Tree cultivated in homegarden	Clearing heat and removing toxicity, promoting blood circulation, lowering blood pressure functions	Medicinal, edible
Yujing Liu, D5 (MUC)	*Liquidambar formosana* Hance	Hamamelidaceae	Feng xiang shu枫香树	Mei yao	Leaves	Cloth	Black	Tree in forest, wild	Expel wind-damp, promote the circulation of qi and detoxify	Medicinal, timber for furniture-making
Yujing Liu, D1 (MUC)	*Polygonum tinctorium* Ait.	Polygonaceae	Liao lan蓼蓝	Bai ya	Stem	Cloth	Dark blue	Herb cultivated in farming field or homegarden	Clear heat and relieve toxicity, cool blood, mainly for curing mumps, decreasing swelling, and relieving pain and itching	Medicinal
Yujing Liu, D9 (MUC)	*Reynoutria japonica* Houtt.	Polygonaceae	Hu zhang虎杖	Xiong shen	Root	Food and cloth	Red	Herb in valley or river side, wild	Expel wind and promote diuresis, scatter stasis, relieve cough and reduce sputum	Medicinal, edible, ornamental, herbal tea
Yujing Liu, D11 (MUC)	*Sassafras tzumu* (Hemsl.) Hemsl.	Lauraceae	Cha mu檫木	**–**	Bark	Food	Brownish red	Tree in forest or mountain slope, wild	None	Medicinal, timber for furniture-making
Yujing Liu, D2 (MUC)	*Strobilanthes cusia* (Nees) Kuntze	Acanthaceae	Ban lan板蓝	Sang mei lan	Leaves	Cloth	Dark blue	Herb cultivated in farming field or homegarden	Clear heat and relieve toxicity, cool blood and relieve sore throat and can kill pathogenic microorganism and improve immunity	Medicinal
Yujing Liu, D12 (MUC)	*Vaccinium bracteatum* Thunb.	Ericaceae	Wu fan shu乌饭树	**–**	Leaves	Food	Black	Tree in forest or mountain slope, wild	None	Medicinal, ornamental
Yujing Liu, D10 (MUC)	*Zanthoxylum simulans* Hance	Rutaceae	Ye hua jiao 野花椒	La san	Root	Food	Red	Shrub in rocky slope, wild	None	Medicinal, spices

"–" means these Dong names are not still used any more. Therefore, we could not record their names. Other Dong names are still used.

## Results

Women are the primary users of dye plants at the study sites and utilize these species to dye customary clothing and food products. Knowledge of species used for dying and prepartion of dyes is orally transferred from mother to daughter. A total of 50, 69, 46, 63, 38, 41, 62, 80, 56, 29 households reported utilizing *Ardisia crenata, Adinandra milletii, Buddleja lindleyana, Buddleja officinalis*, *Dioscorea cirrhosa, Diospyros kaki, Reynoutria japonica, Sassafras tzumu, Vaccinium bracteatum and Zanthoxylum simulans* for coloring food respectively and 43, 31, 49, 80, 36, 51 households reported using *Ardisia crenata*, *Diospyros kaki, Liquidambar formosana, Polygonum tinctorium, Reynoutria japonica and Strobilanthes cusia* for dying cloth. The most frequently reported dye plants were *Sassafras tzumu* and *Polygonum tinctorium*. In addition to dyes, the plants have multiple uses including medicinal, ornamental, sacrificial, edible, and for timber.

The 13 documented plants belong to 9 botanical families (Table 1). The main dyeing use is to color food items followed by dying cloth. A total of 7 species are used for coloring food items, 3 species are used to color clothing and 3 species are used to color both food and clothing. The pigments derived from these plants include black, dark blue, red, brownish red/russet and yellow. In total, 3 of the documented plants are used for black pigments, 3 are used for brownish red/russet pigments, 3 are used for red pigments, 2 are used for dark blue pigments and 2 are used for yellow pigments. Natural dyes are derived at the study sites from various plant parts including leaves (4 species), roots (3 species), flowers (2 species), bark (2 species), stems (1 species) and tubers (1 species).

### Plants used to dye food

**Figure 1:**
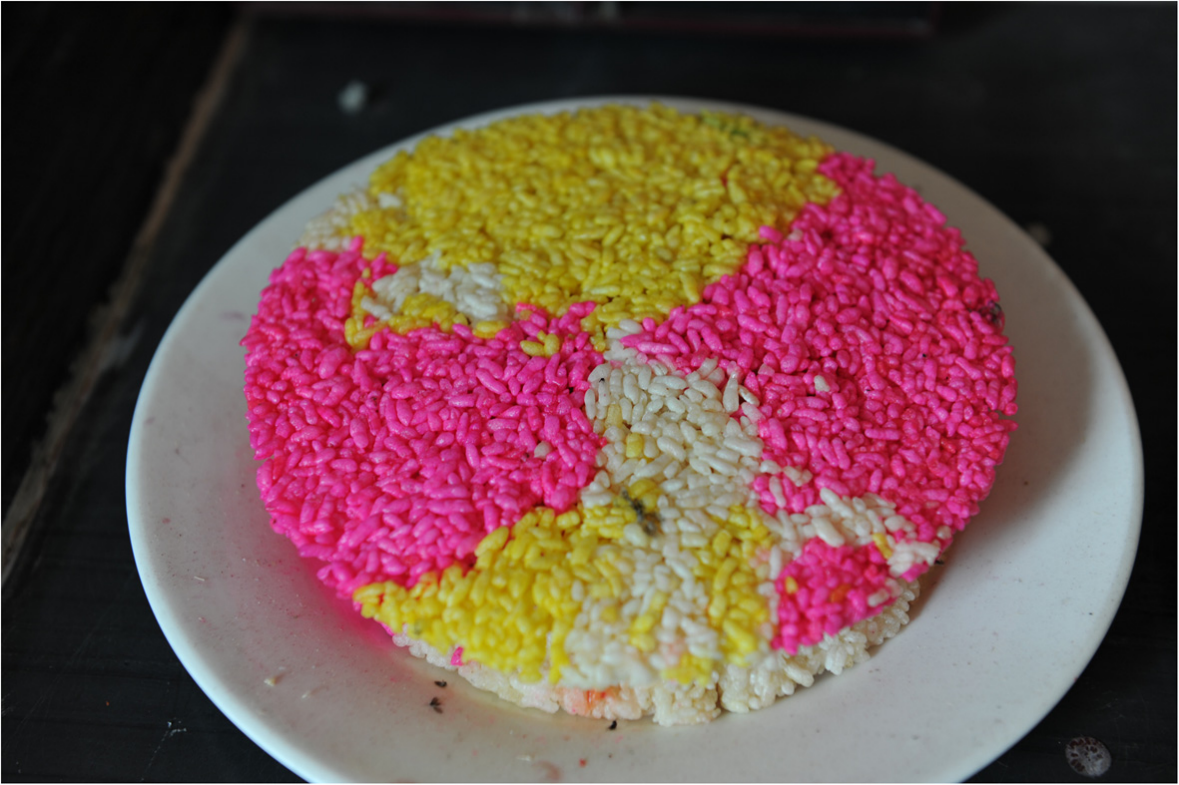
Sticky rice cake dyed by plants in Dong community.

Dong communities have a tradition of eating and dying glutinous rice for daily use, festivals and ritual. Communities use edible plant pigments to enhance their rice with color that invokes traditional beliefs of Dong culture. An example of dyed rice in Dong communities is the colorful glutinous cake known as "festival baba" that is used to celebrate almost all festivals of the Dong people (Figure 1). Festival baba rice cakes are dyed with red, yellow, green and black colors among other colors. Yellow is among the most valued colors for dyeing rice by Dong and is derived from several natural sources. Dong communities primarily derive yellow dye for dyeing rice from *Buddleja officinalis* and *B. lindleyana* (Buddlejaceae). Dye extracted from these plants impart a distinct honey fragrance to rice. Other socio-linguistic groups in China also have a tradition of using this plant to dye rice including the Dai, Zhuang and Yao. Communities harvest the flowers in late spring or early summer. Both dried and fresh flowers can be used for dying. Other plants used to dye food yellow include *Gardenia jasminoides*, *Sophora japonica*, *Curcuma longa*, *Phellodendron amurense*, *Myrica rubra* and *Mahonia fortunei*[7].

A russet brownish red color for food is derived by local communities from the bark of the persimmon tree (*Diospyros kaki*) which is widespread in the study area and contains a brown pigment. Red pigments for coloring rice and other food items in Dong communities are extracted from *Ardisia crenata*, *Reynoutria japonica*, and *Zanthoxylum simulans*, in fact, *Reynoutria japonica* was also recorded before [8]. Dong people use *Vaccinium bracteatum* and *Liquidambar formosana* for a black pigment to dye their rice and other food items.

In addition to adding color to foods, informants perceive that many of their dye plants enhance the edible, medicinal and nutritive value of their food well as impart natural preservatives. For example, the yellow pigment derived from *Buddleja lindleyana* Fortune is locally valued for its medicinal properties including anti-inflammatory, analgesic, calming and liver protecting.

### Plants used to dye clothing

**Figure 2:**
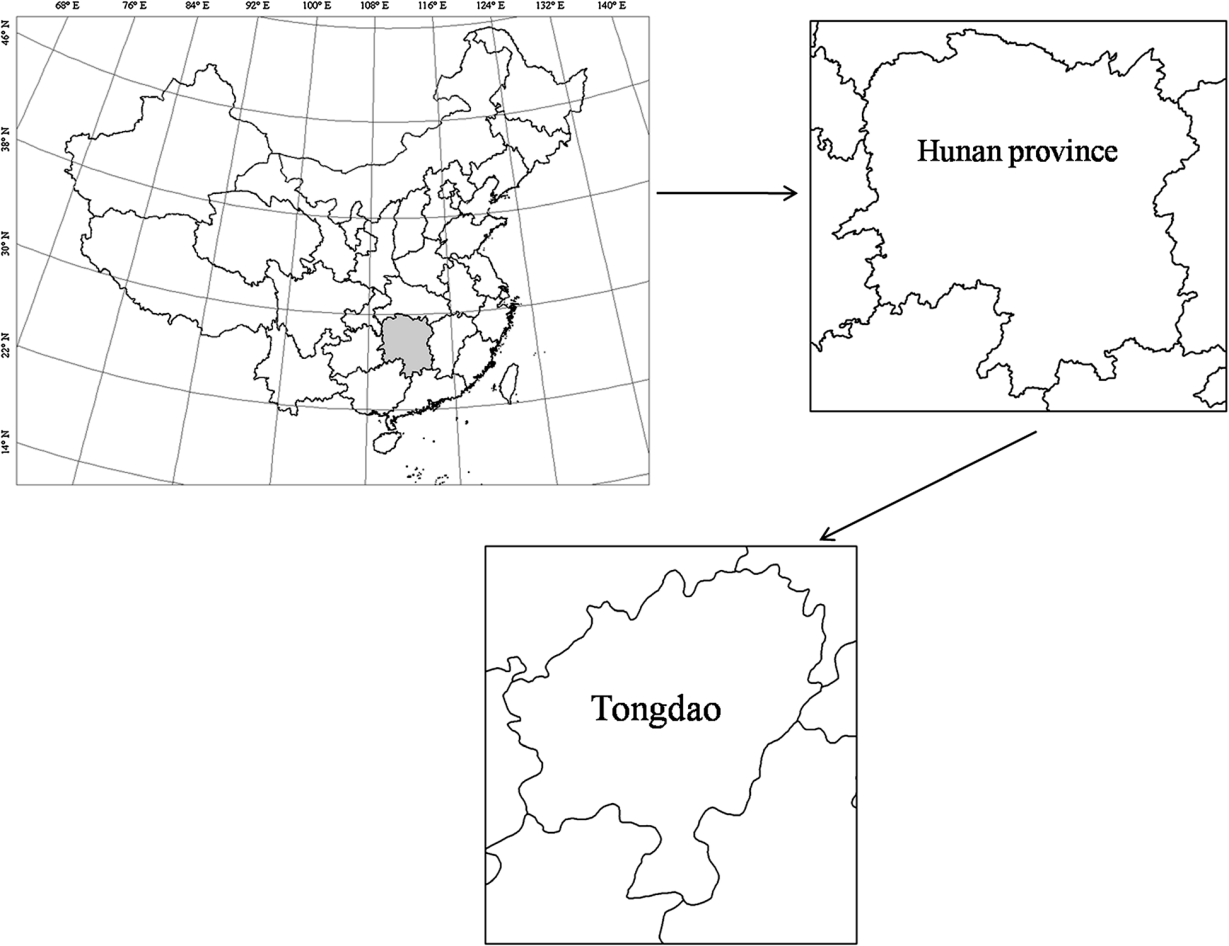
Sketch map of study site.

**Figure 3:**
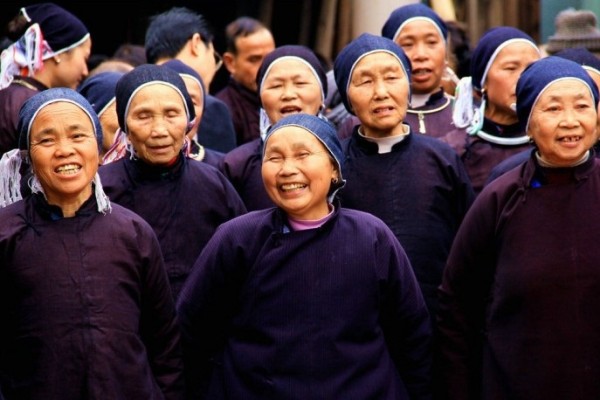
Dong women wearing clothes dyed by plants.

**Figure 4:**
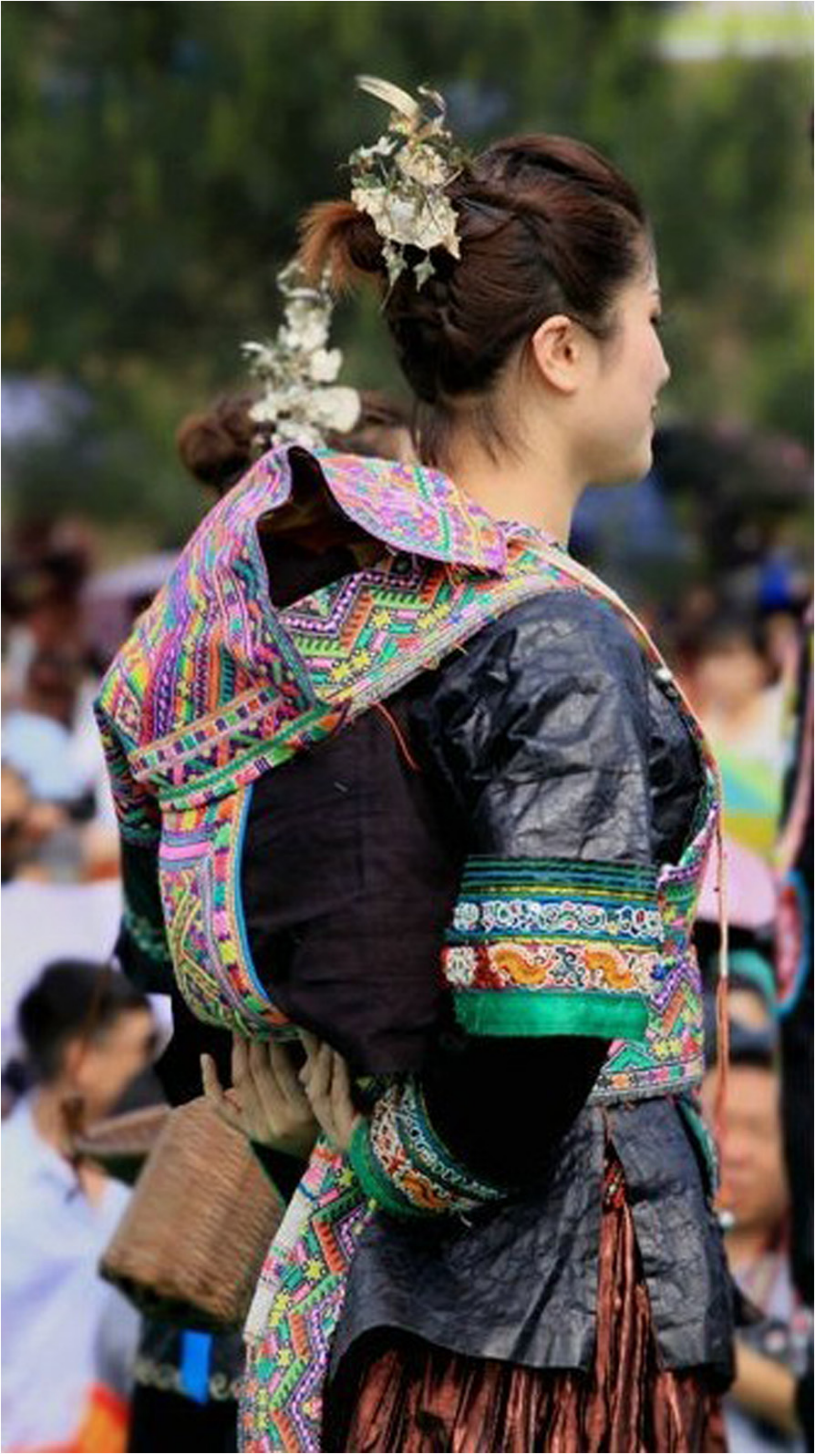
Little Dong girls wearing clothes dyed by plants.

A total of three species were documented that are used to dye the customary clothing of Dong people (Figures 2, 3 and 4) including the dyes known as *ding* (*jinh* in Dong language) and *liang* (*liangc* in Dong language). The dye for *ding* is extracted from either from *Polygonum tinctorium* (Polygonaceae) or *Strobilanthes cusia* (Acanthaceae)*. Ding* results in a dark blue stain that is valued for clothing by Dong informants because it helps hide from prey during hunting. The dye for *liang* is extracted from *Dioscorea cirrhosa* (Dioscoreaceae). The dye *liang* results in a stain that is brownish red and is used by Dong communities to dye the hood of their customary clothing. Local Dong women at the study sites use the flesh of this plant to dye cotton and linen fabric. The dye for black is derived from *Liquidambar formosana* (Hamamelidaceae). In addition to dye food, *Ardisia crenata* and *Diospyros kaki* can dye cloth.

### Traditional processing of dying food and clothing

Dying of food plants involves first drying the dye plant in sun and then mixing the plant with cold water. The plant material is filtered and the resulting extract is added to uncooked food items such as glutinous rice. The mixture is left overnight and then cooked.

There are two general methods of dying cloth in Dong communities. One method is a direct process and the other method involves adding mordants to fix the colors. The direct method is primarily used in Dong areas. The general direct method for dying cloth at the study sites involves the following: place a layer of half-dry *Tetroncium magellanicum* in dye vat followed with a big bunch of straw ash (from rice straw). Fill the dye vat with clean water fetched from river or well to a level that is below the top of the vat by 12–15 cm. Next, pour 4 kg of dried plant dye material such as *ding* and 1 kg of rice wine. Pounding the pigment material and allow to ferment in the vat for 10–12 days. Stir the surface to confirm the dye is ready which will be indicated by the desired color. The dye is then filtered and utilized. Different glossiness can be obtained by utilizing various materials such as wine and ash.

## Discussion

Plant-derived dyes persist at the study sites for their important role in dying food and customary clothing to express Dong cultural identity. The most prevalent use of documented dye plants is to color food, specifically glutinous rice, followed by customary clothing. The pigments derived from dye plants at the study site include red, dark blue, black, yellow and brownish red/russet. These dyes are derived from various plant parts including roots, leaves, flowers, stems, bark, and tubers. In addition to their use as coloring agents, communities value many of these species for their medicinal, ornamental, preservative, edible, and timber uses.

Several of the documented species are recognized for their medicinal value in China. For example, *Polygonum tinctorium* is a species used as traditional Chinese medicine to clear heat and relieve toxicity, cool blood, mainly for curing mumps, decreasing swelling, and relieve pain and itching [9]. *Strobilanthes cusia* is used for its anti-influenza viral activity [10] and to clear heat, relieve toxicity, cool blood, relieve sore throat, kill pathogenic microorganisms and improve immunity [9]. *Buddleja officinalis* is a medicinal beverage recorded in *Chinese Pharmacopoeia*[11] that is sometimes consumed instead of tea in Yunnan Province with a cool and refreshing taste as well as honey fragrance [12]. It is used clinically as a medicine for treating dry eyes caused by liver vacuity, blurred vision and cataracts. It has also shown to have anti-inflammatory and blood glucose-reducing activity and has the ability to strengthen immunity [13-15]. *Buddleja lindleyana* has antibacterial, anti-inflammatory, analgesic calm and liver protecting properties [16]. The persimmon tree (*Diospyros kaki*) is popular in Japan as a medicinal tea with a wide range of pharmacological effects including allaying thirst, clearing heat, removing toxicity, promoting blood circulation and lowering blood pressure functions [9]. It also recognized to have cytotoxic, anti-human immunodeficiency virus (HIV) and anti-*Helicobacter pylori* activities [17]. *Ardisia crenata* is widely distributed in the world and is shown to have many pharmacological functions and clinical applications [18-20] including for curing throat swelling, pain, anti-fertility, antivirus, antineoplastic and insect disinfestation. *Reynoutria japonica* is an antioxidant plant that is used to treat bacteriostasis [8]. In addition, it is used in Traditional Chinese Medicine to expel wind and promote diuresis, scatter stasis, relieve cough and reduce sputum [21]. The black pigment extracted from *Liquidambar formosana* is used to expel wind-damp, promote the circulation of *qi* and detoxify [22].

As society becomes more aware of the toxicity caused by synthetic dyes for both the environment and humans [23,24], plant-derived dyes offer a promising alternative that are gradually attracting global attention [25-28], some ethnobotanical surveys were conducted in Iraq [1], Turkey [2], Argentina [29], Sierra Leone [30] and Peru [31]. Natural plant dyes are particularly appeal to the current trend of sustainability in food and clothing choices. However, it remains difficult to implement plant dyes into industrial production because of their relative low yield, and expensive labor costs. Local small-scale initiatives are feasible to supply the current demand for the high-end niche market of natural dyes and provide a price premium to local communities. Unfortunately, traditional dye resources and their associated knowledge based are being threatened by globalization [28,30]. However, interest in the use of plant dyes has revived due to new market demands and the growth of rural tourism, maybe such traditional knowledge will provide an alternative for the diversification and quality of existing crafts [29].

Traditionally, knowledge of dye plants and their processing in Dong communities was transferred from mother to daughters. As many young Dong people look for jobs outside of their communities, this knowledge base is failing to be transferred as synthetic dyes are become more easily available along with a shift in preference of clothing. Community-based marketing efforts are needed to develop a viable market for the production and sale of dye plants for local communities in order to provide market incentives for their preservation and sustainable use. Future studies are needed to evaluate the phytochemical profiles, bioactivity, stability and safety of plant pigments. In addition, market studies are also needed to develop new uses for dye plants beyond for coloring food and clothing such as for making crafts. These future studies would help to provide guidelines for community-based production and ultimately ethnobotanical preservation.

## Conclusion

The use of dyes derived from plants persists at the study sites for their important role in expressing Dong cultural identity through customary clothing and food. These species are utilized for a range of other uses including medicinal, edible, ornamental, preservative and timber. As globalization and rapid socio-economic change impacts indigenous communities in China, research is needed to investigate these resources and associated culture towards their preservation and sustainable use to support livelihoods.

### Consent

Written informed consent was obtained from the patient’s guardian/parent/next of kin for the publication of this report and any accompanying images.

Permissions were provided by all participants in this study, including the Dong women and girls shown in the photos (Figures 3 and 4). They have declared that they have no objection to the publication of their pictures in the journal. The consent was obtained from the participants prior to this study being carried out. The photographer (Mr. Junhua Wu) transferred the copyrights to the authors.

## Competing interests

The authors declare that they have no competing interests.

## Authors’ contributions

Conceived of the study: YL and CL; Conducted surveys: YL, CL, BL, XW, SL, ZG, JZ, QL; Identified plant species: CL, BL; Analyzed data: YL and SA; Wrote the manuscript: YL and SA; Edited the manuscript: CL, BL, ZG, YL, WH. All authors read and approved the final manuscript.
